# Tissue Engineering and Targeted Drug Delivery in Cardiovascular Disease: The Role of Polymer Nanocarrier for Statin Therapy

**DOI:** 10.3390/biomedicines11030798

**Published:** 2023-03-06

**Authors:** Nunzio Montelione, Francesco Loreni, Antonio Nenna, Vincenzo Catanese, Lucia Scurto, Chiara Ferrisi, Mohamad Jawabra, Teresa Gabellini, Francesco Alberto Codispoti, Francesco Spinelli, Massimo Chello, Francesco Stilo

**Affiliations:** 1 Unit of Vascular Surgery, Università Campus Bio-Medico di Roma, 00128 Rome, Italy; 2 Unit of Cardiac Surgery, Università Campus Bio-Medico di Roma, 00128 Rome, Italy; 3 Residency Program of Vascular and Endovascular Surgery, University of Ferrara, 44121 Ferrara, Italy; 4 Head of Research Unit of Vascular Surgery, Università Campus Bio-Medico di Roma, 00128 Rome, Italy

**Keywords:** statin, polymer, atherosclerosis, cardiovascular disease

## Abstract

Atherosclerosis-related coronary artery disease (CAD) is the leading cause of mortality and morbidity worldwide. This requires effective primary and secondary prevention in reducing the complications related to CAD; the regression or stabilization of the pathology remains the mainstay of treatment. Statins have proved to be the most effective treatment in reducing adverse effects, but there are limitations related to the administration and achievement of effective doses as well as side effects due to the lack of target-related molecular specificity. The implemented technological steps are polymers and nanoparticles for the administration of statins, as it has been seen how the conjugation of drug delivery systems (DDSs) with statins increases bioavailability by circumventing the hepatic–renal filter and increases the related target specificity, enhancing their action and decreasing side effects. Reduction of endothelial dysfunction, reduced intimal hyperplasia, reduced ischemia–reperfusion injury, cardiac regeneration, positive remodeling in the extracellular matrix, reduced neointimal growth, and increased reendothelialization are all drug-related effects of statins enhanced by binding with DDSs. Recent preclinical studies demonstrate how the effect of statins stimulates the differentiation of endogenous cardiac stem cells. Poly-lactic-co-glycolic acid (PLGA) seems to be the most promising DDS as it succeeds more than the others in enhancing the effect of the bound drug. This review intends to summarize the current evidence on polymers and nanoparticles for statin delivery in the field of cardiovascular disease, trying to shed light on this topic and identify new avenues for future studies.

## 1. Introduction

Atherosclerosis (AS) is a chronic, inflammatory disease, affecting the vascular wall. The main factor of this disease is the stockpile of lipids in and on the artery walls. This buildup is called plaque, usually affecting large and medium-sized arteries. Atherosclerosis is a complex and long process [1,2]. It starts damaging the endothelium, followed by the build-up of low-density lipoproteins (LDLs). This leads to various inflammatory processes, catching monocytes, which turn into macrophages. The latter incorporate cholesterols and oxidized LDL, and they become foam cells. The death of these cells triggers the restart of the entire process, leading to a vicious cycle. Any plaque is made up of lipid deposits of foam cells, inflammatory cells, and a fibrous cap covering the plaque [3]. Alongside these processes, another key point in plaque instability and rupture appears to be neoangiogenesis as a result of both ischemia and inflammation. The highly hypoxic environment of the atherosclerotic plaque, due to its necrotic core, paired with the innate immunity cell activation, triggers a cascade that causes the secretion of proangiogenic factors such as vascular endothelial growth factor (VEGF) and basic fibroblast growth factor (bFGF). The resulting blood vessels appear to be dysfunctional and prone to bleeding and rupture, resulting in an increased vulnerability of the plaque itself [1,2,3]. The presence of calcium around the plaque promotes its stability, while its rupture or ulceration leads to instability. The clinical expression of unstable plaque is thrombosis. The rupture of the plaque represents the trigger for the start of the coagulation cascade, which ends with the formation of a blood clot, known as a thrombus, narrowing or blocking arteries [4]. Now, we summarize the most futuristic treatments of atherosclerotic disease with the usage of nanomedicine as a carrier for statins, the main drug in the treatment of dyslipidemias in patients affected by cardiovascular disease. Thanks to nanotechnology, medicine has progressively achieved new ways to diagnose and treat pathologies with a more targeted and precise methods [1,2,3,4]. One of the first applications of nanomedicine has been in the oncology field. The nanoparticles increase the stability, solubility, and absorption of therapeutic drugs, allowing prolonging of the bioavailability. The latter guarantees: (i) the increase in the therapeutic effect of the drug; (ii) protection of the active molecule from enzymatic degradation in tissues or physiological fluids; and (iii) reduction of renal excretion of the active ingredient [5]. Overall, thanks to nanomedicine there is a drastic decrease in side effects caused by drug treatments. In this review, we try to summarize the most futuristic treatment of atherosclerotic disease or the use of nanomedicine as a carrier for statins, the main drug in the treatment of dyslipidemias in patients suffering from cardiovascular disease.

## 2. Search Strategy

For this review, we evaluate all clinical and preclinical studies, investigating the effects on coronary artery disease by statin–nanoparticle complexes. We focused on the most recent studies published (until October 2022) on PubMed, Embase, EBSCO, Cochrane and Web of Science. We used the following keywords in various combinations: (“Hydroxymethylglutaryl-CoA Reductase Inhibitors” (Mesh) OR statin OR statins OR atorvastatin OR rosuvastatin OR lovastatin OR pravastatin OR simvastatin OR *statin) AND (“polymer” (Mesh) OR “nanoparticle” (Mesh) OR “particle” (Mesh)) AND (“atherosclerosis” (Mesh) OR “coronary artery disease” (Mesh)). References of relevant articles were also checked for significant contributions.

## 3. Statins: Summary

Statins represent the main treatment for dyslipidemia, and they have a critical role in the reduction of cardiovascular risk [6,7,8].

**Figure 1:**
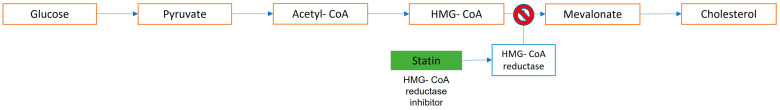
Schematic representation of cholesterol metabolism.

Statins are inhibitors of the HMG-CoA reductase enzyme. This enzyme has a key role in the synthesis of cholesterol in the liver, allowing the conversion of HMG-CoA into mevalonic acid (Figure 1). A high level of glucose allows a better activity of the HMG-CoA reductase, which is indirectly influenced by insulin and glucagon [9]. Statins also have several other effects, such as the improvement of endothelial function, and anti-inflammatory, immunomodulatory, and antithrombotic effects [10]. We highlight that the improvement of endothelial function is mainly driven by the increase in endothelial nitric oxide synthase (eNOS) and stabilization of tetrahydrobiopterin [11,12,13], while the anti-inflammatory effect reduces the production of proinflammatory cytokines.

According to the FDA, the maximum dose of statins is 80 mg/day, except for rosuvastatin, which is 40 mg/day. For example, patients requiring significant LDL reduction should take atorvastatin 80 mg/day or rosuvastatin 20 mg/day or a combination of statin and ezetimibe 10/40 mg/day [14].

The main side effects of statins are: myopathies, muscle inflammation, joint pain, stroke, increased liver enzymes [15,16]. Moreover, the major limitation of these drugs is their short half-life and low bioavailability, leading to a higher dose in order to be effective in patients, however, this increases the risk of side effects. Hence, the newer nanotechnology applications are allowing better management of these drugs by minimizing the side effects and increasing their efficiency.

## 4. Drug Delivery Systems

Nanomedicine is exploiting various carriers and many of them have already been tested and approved. Among these, we have lipid nanoparticles (micelles or liposomes) [17], polymeric nanoparticles [18], and dendrimers [19], and they are summarized in Table 1.

Nanoparticles represent a valuable asset in improving bioavailability and drug efficacy, even besides our topic of interest, statins delivery systems. In recent decades, both organic and inorganic nanoparticles have been widely used and researched in different fields. In current clinical practice, nanoparticle systems are used for gene therapy or drug delivery, especially in antibiotics, cancer, and iron-deficiency treatment [17]. Despite different molecules being popular in new trials, mainly liposomal drug delivery systems are FDA-approved and therefore used in clinical practice, alongside a few polymers and iron-conjugated molecules [18]. New frontiers are represented by lipid particles and polymers (such as PLGA), both being recently approved for clinical use. Lipids are deemed as safe due to their similarities to liposomal structure, hence trials investigating their potential use are easily approved worldwide [17]. Currently, they are used for different drugs such as vaccines, due to the high bioavailability they provide the drugs they are associated with. Polymers are easily synthetized and conjugated to drugs, making them the perfect carrier, due to their biocompatibility and how easily they can be modulated when it comes to stability and responsivity [19]. Such molecules are currently used in clinical practice, mainly combined with chemotherapeutic agents and immunotherapeutic agents, especially due to their ability to escape multidrug resistance. New frontiers are also represented by precision medicines, making nanoparticles a hot topic when it comes to personalized medicine, with hopes to use nanoparticles not only as drug carriers but also as diagnostic tools, paving the way for the concept of theragnostics, where both therapeutic and diagnostic effects are combined [19].

### 4.1. Micelles

Micelles are lipids formed by amphiphilic molecules. In the presence of a water solution, they arrange themselves in a spheric form in order to have the hydrophilic surface in contact with the water, while the hydrophobic one is in the micelle center. Thanks to this structure, micelles are used as carriers for hydrophobic therapeutic agents. They have a diameter of about 10–100 nm.

### 4.2. Liposomes

Liposomes consist mainly of phospholipids that form a double layer that gives them superior biocompatibility. They can incorporate hydrophilic and hydrophobic therapeutic agents. The diameter of the liposome is approximately 40–1000 nm [20]. The importance of liposomes is mainly driven by their low toxicity for in vivo application and by their ability to transfer several molecules.

### 4.3. Polymeric Nanoparticles

Polylactic acid (PLA), poly-glycolytic acid (PGA), and poly-lactic-co-glycolic acid (PLGA) have been approved by the FDA and they have been used for the synthesis of biodegradable nano-DDSs. PLGA is the most common polymer used to treat cardiovascular diseases. Being composed of PLA and PGA, the degradation rate is regulated by the PLA: PGA ratio and this leads to a controlled release of the incorporated drugs. Polymer nanoparticles have a diameter ranging from 20 to 1000 nm. An example of polymeric nanoparticles is leuprolide acetate (a testosterone inhibitor) utilized in prostate cancer (Eligard^®^). PLGA leads to a slow release of leuprolide acetate after subcutaneous injection.

### 4.4. Dendrimers

Dendrimers are made of a central core with external functional groups. Thanks to their internal three-dimensional ramifications, dendrimers are able to incorporate therapeutic agents. Moreover, external functional groups can be substituted by active targets. Their size ranges between 3 nm and 20 nm.

## 5. In Vivo Kinetics of the Carrier System with Nanoparticles

Nano-DDSs have a lot of variables that affect their kinetics, such as geometry, size, and surface. Large nanomaterials (>1000 nm) may build up in the liver and lung and can lead to embolic events in small vessels. Instead, small nanomaterials (<10 nm) present renal excretion. Medium-sized nano-DDSs (10–100 nm) have the best physiologic qualities thanks to their longer bioavailability compared to the previous ones, avoiding renal excretion. Nanomaterials are generally incorporated by the phagocytic mononuclear system (MPS) at the hepatic, splenic, lymph node, and medullary levels [21,22]. The incorporation of nano-DDSs into MPS is one of the mechanisms studied and planned for drug delivery, especially in inflammatory diseases including AS. Another very important aspect is the permeability of target lesions, inflammatory atherosclerotic lesions, or in the ischemic myocardium. Local or systematic inflammation leads to increased vascular permeability with a migration of nanomaterials into the extravascular space.

**Figure 2:**
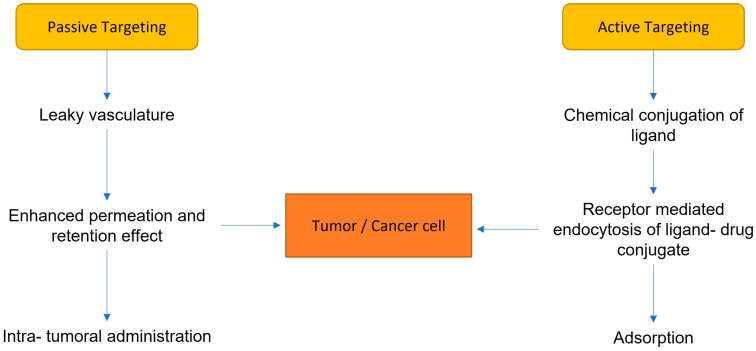
Strategies of targeting.

There are two types of strategies: passive and active targeting (Figure 2). In the oncology field, passive targeting leverages specific characteristics of neoplastic tissue (missing in the physiological one), such as an increase in production of blood vessels and a decrease in lymphatic drainage, in order to store nanoparticles. This mechanism is also called “potential permeability and retention effects” [23]. Instead, the active target is based on exploiting receptors and ligands, such as antibodies or surface proteins on nanomaterials, guaranteeing a high specificity and efficacy of the drug delivery system [23]. To explain active targeting, we can consider angiogenic endothelial cells that express specific receptor molecules that are either not expressed or are only rarely expressed on normal endothelial cells. This allows for the design of targeted nanoparticles. Selective ligands for these receptors are considered useful for targeted drug delivery to angiogenic vessels. The peptide Ala-Pro-Arg-Pro-Gly (APRPG) has been identified as the best ligand targeted to angiogenic vessels through the VEGF-1 receptor [23]. APRPG-modified liposomes (APRPG liposomes) have been developed for targeted drug delivery to angiogenic vessels. The tissue biodistribution of APRPG liposomes after intravenous injection was enhanced, and PEG liposomes modified by APRPG (APRPG–polyethylene glycol (PEG) liposomes) were developed to increase their half-life in the bloodstream and the possibility of binding to angiogenic vessels [23].

## 6. Biological and Clinical Effects

**Figure 3:**
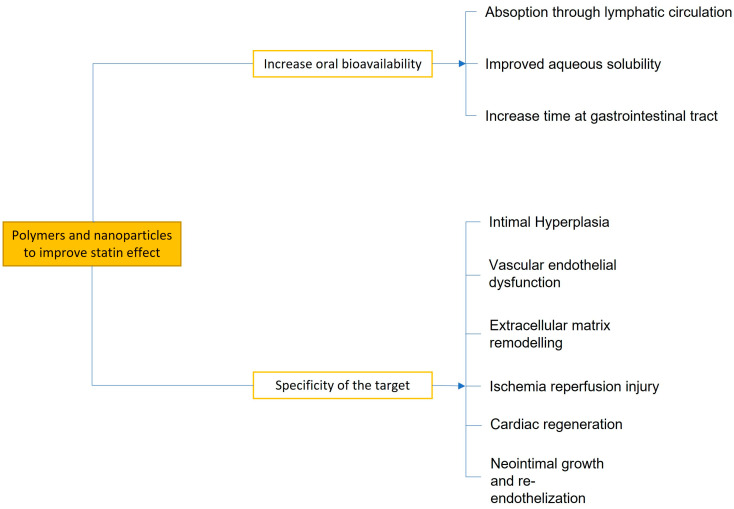
Summary of the main effects of polymer-based strategies for statin delivery.

Conjugated statins + nano-DDSs can improve the therapeutic effects, leading to a tight management of coronary disease thanks to their higher bioavailability and greater specificity. We analyze, through the most recent studies published, the improvements that may occur using the statin–nanoparticle complex (Figure 3).

Nanoparticles are recognized in the scientific community as the new frontier in target therapy. Despite not yet being approved in statin delivery, they appear to be a promising asset in future cardiovascular disease treatment. From preliminary studies, nanoparticles use could not only increase drug bioavailability but could also allow reduction of drug dosage in patients, lowering side effects associated with statins. As previously stated, both lipid-based and polymeric carriers represent a potentially key asset in drug delivery, and statin therapy does not constitute an exception, hence our interest in the subject. Nanoparticle use could also increase therapy specificity, making cardiovascular disease treatment more patient-tailored.

### 6.1. Bioavailability

Statins are marketed as tablets to be taken orally. Usually, oral administration leads to several disadvantages, such as the low solubility, rapid metabolism, and low bioavalaibility, reducing the drug’s clinical effects [24]. Bioavailability ranges from 5% (simvastatin) [25] to 20% (rosuvastatin) and on average 12% for the most commonly prescribed compound (atorvastatin) [26,27] (Table 2). Thanks to new strategies, the bioavailability has been improved, leading to an increase in the permanency of drugs through the gastrointestinal tract. One of the main functions of the lymphatic system is the absorption of nutrients, such as fats, or drugs taken orally [28]. It has been described how some nanoparticles are able to reach the lymphatic system of the intestine, managing to evade the enterocytic enzymes and the first hepatic degradation [29]. Thanks to their mechanical properties and good stability in different physiological pH ranges [26,27,28,29,30], cellulose-based polymers [27] guarantee a slow release of the drug, increasing its bioavailability. Cellulose-free polymers, polyoxypropylene and polyoxyethylene, lead to a four-fold increase in the bioavailability of atorvastatin [26], but they should be carefully studied for their variability and size. Ethylcellulose unifies the advantages of biodegradability and biocompatibility with a considerable cost–benefit ratio for large-scale use [30,31] and it is the most promising as a carrier for oral statins. Recently, some authors have described how atorvastatin with Pluronics^®^ (amphiphilic biopolymers) can increase the bioavailability of the drug and make it highly selective, further extending the effects of the drug [27] (Table 3).

### 6.2. Endothelial Dysfunction

**Figure 4:**
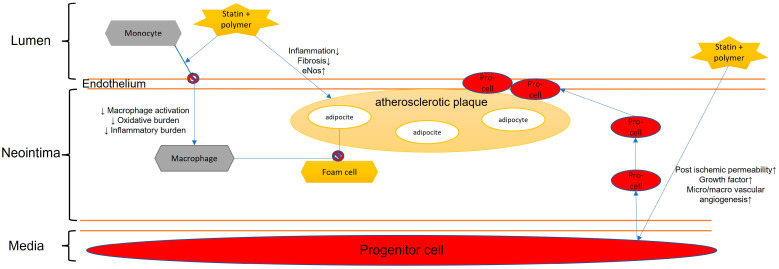
Statin role in endothelial dysfunction.

Associated with aging and cardiovascular diseases, endothelial dysfunction is a key process in atherosclerosis [32] (Figure 4). The treatment remains debated and complex due to the deficiency of different drugs targeted to the endothelium’s cells. Nanoparticles, through endocytic pathways, have enabled new pharmacological treatments [33]. Vascular endothelium dysfunction is a common pathway of many altered transduction systems, such as nitric oxide synthase, ROS production, etc. [34,35]. In order to reduce oxidative and inflammatory stress, decreasing macrophage activation, poly-di-methyl-siloxane and poly-2-methyl-oxazoline vesicles are loaded with pravastatin [36]. It has been highlighted how simvastatin, combined with nanoparticles, brings different positive effects: (i) it reduces macrophage multiplication in atherosclerotic plaques [37], (ii) it blocks the activation of TNF-alpha and interleukin 1 beta [38], (iii) it prevents the synthesis of mevalonate, encouraging a positive endovascular remodeling [39]. PLGA, coupled to pitavastatin, inhibits monocyte activation through suppression of chemotactic proteins and stimulating factors, therefore reducing inflammation [40,41].

Recent studies have examined highly selective administrations of atorvastatin targeted to activated macrophages, thanks to the binding of nanoparticles on the macrophage membrane. Hence, the drug is not susceptible to reticuloendothelial clearance. Targeted atorvastatin reduces the instability of the atherosclerotic plaque thanks to the reduction of metal proteases, the increased production of collagen, and the improvement of the lipid profile [42]. PLGA + statin guarantees inflammation reduction and consequently increases the therapeutic effect of the molecules [43]. This nanoparticle-based biomimetic approach represents an interesting option for future studies as it could actively modify the pathological microenvironment and improve vascular endothelial function.

### 6.3. Intimal Hyperplasia

One of the main aspects of atherosclerotic pathology is intimal hyperplasia, which is a consequence of the inflammatory state [44]. Statin treatment reduces intimal hyperplasia by approximately 25%. Nanoparticles, especially polysialic acid–polycaprolactone combined with simvastatin, were able to significantly reduce the intimal hyperplasia of vascular smooth muscle cells [45] (Table 3). This was mainly driven by the stimulation of nitric oxide synthase, producing NO, and the reduction of isoprenoids [46,47]. 

### 6.4. Neoangiogenesis

Neoangiogenesis represents a key point in plaque instability, due to the proliferation of highly irregular dysmorphic vessels in atherosclerotic plaques. Growing evidence suggests that plaque neovascularization is an important contributor to plaque growth and instability [48,49]. Plaque neoangiogenesis is a physiological response to the increased oxygen demand, and the molecular mechanism is mainly driven by hypoxia-inducible factor (HIF) and inflammatory mediators, with a feedback cycle between hypoxia, neoangiogenesis, and extravasation of red blood cells, leucocytes, and plasma lipids [48]. The vessels undergoing neoangiogenesis have a significant degree of immaturity, with structural and functional abnormalities, such as a discontinuous basal membrane or a reduced number of tight junctions between the endothelial cells [49]. This leads to vessel destabilization and increased permeability, favoring leakage of lipids and cells into the interstitial space [49]. On the other hand, oxLDL, proteases, and products of oxidative stress enhance extracellular matrix damage, resulting in intraplaque hemorrhage [48]. Those events create a milieu of local oxidative stress with sustained inflammatory activity, that might increase the risk of plaque rupture.

Neoangiogenesis is frequently associated with thromboembolic events and plaque rupture [48,49]. This may suggest that antiangiogenic therapies may be an asset in plaque-stabilizing protocols. Systemic anti-VEGF therapies and other antiangiogenic agents, although having been used in some oncological experimental protocols, appear to be linked with a high risk of arterial thromboembolic events [50]. Due to such contraindications, preliminary studies where anti-VEGF drugs are used combined with nanocarriers have recently emerged. In the oncological field, among anti-VEGF drugs, bevacizumab is widely known as a valid option for targeted therapy in highly vascularized tumors. This recombinant humanized monoclonal antibody has been tested in neuroblastomas, showing its effects against neoangiogenesis. According to new evidence, carriers already used in statin delivery, such as bevacizumab, could be used for targeted delivery, allowing targeted therapy without systemic adverse effects. In a preliminary study [51], ανβ3 integrin-targeted paramagnetic nanoparticles that incorporated fumagillin at 0 μg/kg or 30 μg/kg were administered to cholesterol-fed rabbits, showing promising results in vascularization decrease at 1-week MRI. Further experiments analyzed potential synergy between this protocol and statin administration in the same population [52], confirming the effects these combined drugs have on reducing plaque neoangiogenesis, increasing its stability. Such results, despite being in a preclinical phase, could lead to a breakthrough in cardiovascular therapies, with nanocarriers being used not only as statin carriers but as multidrug carriers with wide effects on plaque stabilization.

### 6.5. Ischemia–Reperfusion Injury

Reperfusion injury plays a dramatic role in myocardial ischemia [53]. Statin administration was associated with a reduction in myocardial ischemic zone, via the PI3K/Akt pathway and RISK [54] kinases, but it has not yet been fully confirmed in preclinical and clinical studies due to the lack of highly selective compounds towards the cardiac ischemic zone [55]. This problem has been overcome thanks to PLGA, a nanoparticle able to reach the ischemic tissue thanks to its vascular permeability characteristics [18]. In a preclinical model of myocardial infarction, intravenous administration of these nanoparticles, loaded with pitavastatin, allowed them to selectively reach the area damaged by ischemic–reperfusion injury, reducing the myocardial infarction zone and enhancing left ventricular performance [56].

### 6.6. Cardiac Regeneration

After a myocardial ischemic event, “neocardiomyogenesis” allows a limited generation of myocardial cells. Statins can increase the regenerative potential of cardiac cells. In a model of chronic myocardial infarction in rats, pravastatin, simvastatin, and rosuvastatin are able to amplify cardiac stem/progenitor cells by stimulating their differentiation towards the myogenic lineage [57]. The use of simvastatin-associated PLGA on ischemic myocardium of mice improves cell migration and growth factor expression, inducing endogenous cardiac regeneration. The regenerative capacity must be traced back to the fat-derived stem cells, in which simvastatin exerted a crucial effect. After 1 month, the granulation tissue of the chronic infarction was largely replaced by regenerated myocardium, reducing the fibrosis of the ischemic scar. Research is mainly focusing on adipose-derived stem cells due to their plasticity, easy collection with minimally invasive procedures, and their ability to differentiate into cardiomyocytes [58,59]. The use of statins could contribute to the differentiation of cardiomyocytes after an ischemic event. Hence, polymers, loaded with statins, represent an excellent and innovative therapeutic approach for CAD.

### 6.7. Remodeling in the Extracellular Matrix

The outcome of coronary heart disease leads to a disruption of the extracellular matrix during the inflammatory and reparative phase [60]. Nanoparticles, loaded with pitavastatin, are able to protect the extracellular matrix from postischemic remodeling, by blocking the migration of monocytes [61,62]. This confirms that the targeted statins can improve the healing process of the heart area affected by ischemia [63,64]. PLGA seems particularly suitable thanks to its easy degradation, its prolonged release of the drug, and its very high biocompatibility [65]. Hence, the pitavastatin–PLGA complex has been proposed as a therapeutic strategy for postischemic remodeling of the left ventricle in the case of CAD [61,62,63,64,65,66] (Table 4). Other studies have reported atorvastatin-induced improvement in left ventricular remodeling after acute myocardial infarction, through the interaction with collagen metabolism, such as with matrix metalloproteinases [67,68].

### 6.8. Neointimal Growth and Reendothelialization

Percutaneous procedures (PCIs), with coronary stent implantation, have become a key treatment for acute coronary syndrome and, in some particular cases, for chronic angina. Medicated stents have gained a lot of ground in reducing intrastent restenosis (ISR). ISR occurs in 25% of patients undergoing PCIs, caused by neointimal hyperplasia. Therefore, bare metal stents have been gradually replaced by drug-eluting stents such as everolimus and sirolimus, which have shown reduced risk of ISR [69,70,71]. Statins were examined considering their effect on the endothelium, such as (i) the inhibition of intimal hyperplasia or cell proliferation, (ii) the inhibition of the mTOR pathway, through the activation of AMP-K, and (iii) the inhibition of the inflammatory cascade carried out by macrophages. MTOR is implicated in ISR, through many signaling pathways. Statin-eluting stents could play a role in the prevention of ISR in PCIs [72]. However, this new method has not yet been adequately tested, but state-of-the-art studies are delivering significant results, such as that by Jin Chu et al. who studied atorvastatin-medicated stents to promote neoendothelium formation [73].

### 6.9. Anti-Inflammatory Target Therapy

Nanocarriers can be used in targeted delivery of anti-inflammatory agents such as interleukins, especially IL-10. While IL-10 systemic administration has appeared to not be linked to significant reduction in serum levels of cytokines, its targeted release when linked to PLGA appears to induce cap fibrosis and thickness and to reduce the necrosis percentage in the plaque core, inducing plaque stability [74,75]. Preliminary studies showed encouraging results in inflammation reduction when using statins as targeted therapy. According to Leal et al. [76], PLGA can be used as a carrier in delivering atorvastatin combined with miRNA-124a, drastically reducing the levels of proinflammatory cytokines such as TNF-α and IL-6, and of reactive oxygen species (ROS), in LPS-activated macrophages and vessel endothelial cells.

## 7. Clinical Implications

The efficacy of statin therapy to improve cardiovascular morbidity and mortality is firstly related to its ability to lower LDL values, leading to a reduction of 12% of global deaths per reduction of LDL cholesterol mmol/L [77]. Most of the effect of statin therapy seems to be produced by the relative reduction in LDL cholesterol values rather than by the achievement of an absolute LDL target level. Thus, statins are always recommended in ischemic heart disease, regardless of baseline LDL cholesterol levels [78]. A high-dose strategy was shown to be more effective than a low-dose one in preventing death, keeping an acceptable safety profile [77]. Additionally, the clinical literature shows the potential alteration in bleeding risk of chronic statin treatment related to alterations in angiogenesis, platelet function, and coagulation cascade [79,80,81,82,83]. Those features should be carefully investigated with the use of polymers as the side effects can be dramatically reduced by targeted therapy.

### 7.1. Biological Evidence about Statins and Angiogenesis

Angiogenesis can be either a physiological or a pathological process involved in the pathogenesis of ischemia, chronic inflammation, and tumors [84]. A critical role in angiogenesis is played by VEGF, a growth factor mostly induced by hypoxia-inducible factor (HIF) and down-regulated by the von Hippel–Lindau (VHL) suppressor gene. It stimulates angiogenic response in in vivo and in vitro models, bringing survival and migration signaling to endothelial cells (ECs) and inhibiting their apoptosis [85]. Currently, we know three different receptors of this factor, each one with many different properties. While VEGFR1 is known for its inhibitory activity on VEGF signaling, VEGFR2 relates to the most known angiogenic properties of this factor. It is primarily activated by the so-called “canonical ligands”, e.g., its natural activator VEGF-A and co-receptors such as neuropilin (NRP1 and NRP2) and heparan sulfate proteoglycans (HSPGs). Besides these, there are many “non-canonical ligands”, which can stimulate angiogenesis by activating VEGFR2 in mechanical shear stress, such as gremlins, galectins, lactate, and LDLs [85]. Evidence shows that statins may interfere with VEGF expression. Frick et al. have shown that this interference may be strictly related to the dose of statins and the type of cells. They studied three different cell populations (endothelial cells derived from human umbilical cord veins—HUVECs; human vascular smooth muscle cells—HVSMCs; microvascular endothelial cells—HMEC-1) and their constitutive expression of VEGF. Different statins (atorvastatin, simvastatin, and lovastatin, 1–10 mol/L) significantly reduced basal VEGF synthesis in HMEC-1 and HVSMCs and, at the same doses, up-regulated VEGF generation in HUVECs. In this last cell type, at low concentrations (0.03–1 micromol/L) the proangiogenic activity was prevalent, whereas, at higher concentrations, the antiangiogenic activity increased, despite a concomitant increase in VEGF synthesis [86]. Other authors collected blood samples from 14 male hypercholesterolemic patients with angiographically confirmed CAD at baseline and after two months of atorvastatin (20 mg/day) therapy. Then, human coronary artery smooth muscle cells (HCASMCs) were incubated with plasma derived from both samples. Atorvastatin reduced serum VEGF levels in CAD patients. Additionally, the plasma collected before therapy, compared to plasma collected after treatment and controls, induced a significantly higher amount of VEGF in HCASMCs [87]. Recently, in an atherosclerotic animal model of type 2 diabetes mellitus, simvastatin has been shown to significantly reduce VEGF and increase TGF-beta1 expression in 4–8 weeks. Interestingly, it seemed that simvastatin was able to reduce VEGF almost exclusively during the first week [88].

In addition to their effects on VEGF expression, statins are known for many other antiangiogenic properties. In 2002, Weis et al. demonstrated that statins tend to biphasically influence angiogenesis in a dose-dependent manner. Both in vivo and in vitro, high-dose atorvastatin (0.05 to 1 micromol/L) inhibited EC proliferation, migration, VEGF and VEGFR2 expression, and inflammation-induced angiogenesis. Opposite results were obtained with low-dose atorvastatin (<0.01 micromol/L) [89]. Schaefer at al. showed that in HUVECs cerivastatin, simvastatin, and fluvastatin caused a dose-dependent inhibition of EC growth. The authors reported that cell proliferation induced by oxLDL (10 μg/mL) could be effectively prevented by using statins at concentrations between 0.01 and 0.1 μmol/L (cerivastatin), 1 and 2.5 μmol/L (simvastatin), and 0.25 and 1 μmol/L (fluvastatin). Their study demonstrated that statins can directly inhibit oxLDL-induced angiogenesis [90]. Again, it has been demonstrated that in HUVECs and vascular endothelial cells different statins may inhibit angiogenesis dose-dependently by inducing the mitochondrial pathway of apoptosis [91,92].

### 7.2. Statins and Bleeding Risk

Despite the efficacy of statins being investigated in many RCTs and clinical studies concerning their use in primary and secondary prevention of ischemic and thrombotic events, very little is known about their influence on bleeding risk. Theoretically, every molecule which demonstrates antithrombotic properties should increase the supposed bleeding risk. Differently from other medications (such as antiplatelets or anticoagulants), whose antithrombotic effects are balanced by increased chances of blood loss, statins do not seem to show these properties.

In the SPARCL trial, high-dose atorvastatin reduced ischemic stroke incidence by 2.5% in 4731 patients with previous stroke or TIA within one to six months before being enrolled, with LDL cholesterol levels of 100 to 190 mg/dL, and without known coronary heart disease. In the atorvastatin group, these effects were balanced by a small but significant increase in hemorrhagic stroke risk, compared to placebo [93]. Increased incidence of intracerebral hemorrhage (ICH) in patients undergoing statin therapy seems to be directly related to the LDL cholesterol value reduction rather than their antithrombotic properties. The biological reasons for this effect are not completely known, but the role played by LDL in the cell membrane’s integrity is likely to be involved [94]. A large prospective study, with more than 90,000 patients enrolled, showed that LDL values below 70 mg/dL could be directly related to increased ICH risk, independently from other risk factors, such as age, sex, BMI, hypertension, or alcohol intake [95]. A recent meta-analysis of 43 different studies (including the SPARCL trial) has considered two different groups of patients: “previous ischemic stroke” and “previous ICH”. In the “previous ICH” group, statins did not increase the risk of ICH recurrence and significantly reduced all-cause mortality (RR 0.49, 95% CI 0.36 to 0.67, *p* < 0.001). In the “previous ischemic stroke” group, statins still increased, but not significantly, ICH risk (RR 1.61, 95% CI 0.77 to 3.34; *p* = 0.20) and reduced all-cause mortality (RR 0.68, 95% CI 0.50 to 0.92, *p* = 0.01). Despite this, the authors reported how studies with more robust design and lower bias risk (mostly RCTs) were the ones that showed the strongest association between statins and ICH risk [96]. Currently, it is not clear whether a real increased ICH rate exists or whether it is related to statin therapy or low LDL values (or both). This is a critical matter in terms of statins’ safety profile. Nonetheless, it still seems far from being completely clarified. Recently, Ribe et al. have published a cohort study deriving information from 5 Danish registers, evaluating the ICH risk in statin users and non-users in 5,519,894 stroke-free individuals for ten years of follow-up. Surprisingly, this study showed that ICH risk was reduced by 22–35% in statin users compared to statin non-users after the first six months of therapy. During the first six months, ICH risk was similar in both groups [97]. Due to this conflicting evidence, it is not possible at this time to give any definitive conclusion about statin use and ICH risk. As reported in the latest ESC guidelines, there could be concern about statins’ safety profile regarding ICH, but the benefit on the global stroke risk surely justifies their use in this setting as supported by the most recent evidence [98].

It is uncommon for patients with many cardiovascular risk factors to simultaneously use statins and oral anticoagulants (OACs) in primary or secondary prevention of ischemic stroke and VTE. In some populations, up to 40% of warfarin users are concomitantly treated with statins [99]. These subjects are exposed to a greatly increased bleeding risk, and it could be detectable in those patients if statin therapy were significantly associated with increased bleeding rate. Some studies tried to answer this question. A monocentric cohort study of 1686 patients under warfarin treatment found that rosuvastatin was associated with an increased risk of gastrointestinal (GI) bleeding [100]. Schelleman et al. have hypothesized that increased GI bleeding and ICH incidence in warfarin-treated patients who use statins could be restricted to those molecules sharing cytochrome metabolism (CYP3A4) [101]. However, this is questioned by other authors. As reported in a prospective study of 738 patients under phenprocoumon or acenocoumarol treatment, concomitant therapy with rosuvastatin or simvastatin led to a significant vitamin K antagonist (VKA) dose reduction in order to keep acceptable INR values. This could be only partially explained by a pharmacokinetic interaction since rosuvastatin shares only 10% of its metabolism with VKAs [102]. Evidence about GI bleeding incidence and statin therapy is extremely contradictory and different studies show different or even conflicting results. In 2015, a retrospective study divided 12,000 patients into two subgroups defined as statin users (use for at least 90 days) and non-users (never received statin). After a propensity score matching based on 82 variables, no significant interactions between statin consumption and incidence of GI bleeding were found [103]. In the specific ischemic setting, a retrospective study of the OPUS TIMI 16 trial has been conducted to find how statins influenced GI bleeding risk. In this trial, 10,288 patients with ACS who underwent coronary angiography (all treated with aspirin) were randomized to placebo vs. orofiban (Gp IIb/IIIa inhibitor) groups. The authors evaluated the effects of statin therapy in both groups. After 10 months of median follow-up, 1% of patients treated with statins suffered GI bleeding against 1.8% of patients who were not taking statins (P = 0.001). Statins reduced GI bleeding incidence in both orofiban and placebo groups [104]. There are few studies regarding statin-related bleeding risk in specific clinical settings where they have demonstrated antithrombotic efficacy. In 2015, Riva et al. published a retrospective and multicentric study in 681 patients treated with VKAs in the secondary prevention of VTE. Bleedings were divided into “major bleedings” (“MBs”, according to ISTH classification) and “clinically relevant non-major bleedings” (“CRNMBs”, defined as all bleedings requiring medical attention and/or therapy withdrawal and not matching MB criteria). Multivariate analysis showed that statins were not associated with an increased MB or CRNMB risk [105].

### 7.3. Limitations

In addition to coronary artery disease, nanotechnology can be applied to a wide variety of conditions such as pulmonary hypertension, prevention of venous graft stenosis, and coronary intrastent stenosis. Early clinical trials have already been completed to test the efficacy of drug delivery systems with statins in patients with critical limb ischemia [21]. Of course, at the current stage it is not possible to clearly elucidate all beneficial and harmful effects, despite the promising results of pioneering studies. The only limitation shared by the literature is the poor duration of follow-up and the youth of this innovation; also, the quality and cost of nano-DDSs, with a highly complex technology to realize, remain a significant concern that will be solved by future studies.

## 8. Conclusions

Advanced atherosclerotic plaques have several specific features, such as neovascularization, microcalcification, cholesterol accumulation, and cell necrosis, that can lead to plaque destabilization and clinically significant cardiovascular events. These mechanisms strongly depend on the lipid content and local inflammatory events.

Statin treatment represents a milestone, but the effects of systemic administration could be enhanced by targeted delivery of statins, through polymers and nanoparticles [76].

Different nanoparticles have already been approved by the FDA. However, their applications can vary based on their characteristics, such as bioavailability, size, and geometry. We analyzed different processes affecting atherosclerotic pathology and the positive effects of using statin nanoparticle complexes. The administration of statins via PLGA appears to be the most promising aspect of current research due to its easy degradation, its prolonged release of the drug, and its biocompatibility. In the case of endothelial dysfunction, PLGA, coupled with a statin (such as pitavastatin), reduces the inflammatory process through the inhibition of monocyte activation. In myocardial ischemia, PLGA is also able to reach the ischemic tissue, reducing the myocardial infarction zone thanks to its vascular permeability characteristics. In the “neo-cardio-myogenesis process”, the PLGA–simvastatin complex also guarantees cell migration and growth factor expression, inducing endogenous cardiac regeneration. Moreover, as anti-inflammatory targeted therapy, PLGA can be used as a carrier, reducing the levels of proinflammatory cytokines. Several studies have also highlighted the use of other polymers and nanoparticles as alternative treatments. Statins, such as simvastatin, combined with poly-di-methyl-siloxane and poly-2-methyl-oxazoline, reduce inflammation and endothelial dysfunction. A preliminary study on neoangiogenesis, a key point in plaque instability, showed that the use of ανβ3 integrin-targeted paramagnetic nanoparticles reduces neovascularization in rabbits. Moreover, metalloproteinases have shown an important role in the remodeling of the left ventricle, after acute myocardial infarction.

Overall, several preclinical studies have highlighted the effect of statins in the stimulation and differentiation of endogenous cardiac stem cells, as well as in targeting local adverse conditions implicated in atherosclerosis. The administration of statins via PLGA appears to be the most promising aspect of current research due to its advantages in different paths. Future studies will reinforce this hypothesis and clarify the clinical role of polymer-based statin administration in reducing cardiovascular events in patients with coronary artery disease.

## Figures and Tables

**Table 1:** Potential drug delivery systems for statin release.

	Micelle	Liposome	Polymer Nanoparticle	Dendrimer
Geometry	Vesicles formed from a monolayer of synthetic lipids or amphiphiles	Vesicles formed by double layers of phospholipids	Formation of macromolecular polymers	Highly branched macromolecules from a central nucleus
Size (nm)	10–100	40–1000	20–1000	3–20
Features	Incorporation of hydrophobic agents inside	Encapsulation of hydrophilic agents, incorporation of hydrophobic agents into the membrane	Incorporation of hydrophilic and hydrophobic agents with controlled release of embedded agents	Multivalent properties by exterior function groups

**Table 2:** Chemical differences among currently used statins.

Parameter	Atorvastatin	Simvastatin	Rosuvastatin	Pitavastatin	Pravastatin
Prodrug	No	Yes	No	No	No
Hydrophilic	No	No	Yes	No	Yes
Fraction Absorbed (%)	30	70	Unknown	80	34
Bioavailability (%)	12	5	20	80	18
Active Metabolites	Yes	Yes	Yes	No	Yes
Half-Life (hours)	15–30	2–3	20	11	1.5–2.5
Hepatic Metabolism (%)	70	78–87	63	Unknown	45–65
Renal Metabolism (%)	2	13	10	2	60

**Table 3:** Effects of different nanocarriers for statin delivery.

PLGA	Poly-di-mehyl-siloxane Poly-2-2methyl-oxazoline	Nanoliposomes	Polysialic Acid–Polycaprolactone
↑ Chemotactic proteins	↓ Macrophage activation	↓ Isoproterenol	↓ Nitric oxide
↑ Postischemic permeability	↓ Oxidative burden	↓ Fibrosis	↓ Rho pathway
↑ Growth factor	↓ Inflammatory burden	↓ Inflammation	
↑ Micro/macrovascular angiogenesis			
↓ Monocyte mobilization			

**Table 4:** Effects of statin-loaded nanocarriers in the extracellular matrix.

Drug	Carrier Type	Diameter (nm)	Targeted Cell Types/Receptors	Outcomes Compared to Free Drug	Superior Compared to Free Drug
Pitavastatin	PLGA	196	Alveolar macrophages, smooth muscle cells	eNOS ↑ (40%), NF-κB ↓ (60%) Smooth muscle cells ↓ Pulmonary hypertension ↓ Survival ↑ (20%) Chemotactic proteins ↑ Post ischemic permeability ↑ Growth factor ↑ Micro/macrovascular angiogenesis ↑ Monocyte mobilization ↓	Yes
Simvastatin	PLGA	233	VCAM-1		No
Simvastatin	DSPC, DSPG, cholesterol	164	Macrophages, monocytes	Monocytes ↓ (24%) Stenosis ↓ (33%)	Yes
Simvastatin	rHDL	26	Macrophages, endothelial cells	Plaque area ↓ (36%), macrophages ↓ (84%) Inflammation ↓ Fibrosis ↓	Yes
Pravastatin	PDMS/PMOXA	97	SR-A1	LDL uptake ↓	Yes

## Data Availability

Not applicable.
